# The Role of the Mu Opioid Receptors of the Medial Prefrontal Cortex in the Modulation of Analgesia Induced by Acute Restraint Stress in Male Mice

**DOI:** 10.3390/ijms25189774

**Published:** 2024-09-10

**Authors:** Yinan Du, Yukui Zhao, Aozhuo Zhang, Zhiwei Li, Chunling Wei, Qiaohua Zheng, Yanning Qiao, Yihui Liu, Wei Ren, Jing Han, Zongpeng Sun, Weiping Hu, Zhiqiang Liu

**Affiliations:** 1 MOE Key Laboratory of Modern Teaching Technology, Shaanxi Normal University, Xi’an 710062, China; duyinansnnu@snnu.edu.cn (Y.D.); ykz.mtt@snnu.edu.cn (Y.Z.);; 2 School of Psychology, Shaanxi Normal University, Xi’an 710062, China

**Keywords:** acute restraint stress, stress-induced analgesia, mu opioid receptor, medial prefrontal cortex

## Abstract

Mu opioid receptors (MORs) represent a vital mechanism related to the modulation of stress-induced analgesia (SIA). Previous studies have reported on the gamma-aminobutyric acid (GABA)ergic “disinhibition” mechanisms of MORs on the descending pain modulatory pathway of SIA induced in the midbrain. However, the role of the MORs expressed in the medial prefrontal cortex (mPFC), one of the main cortical areas participating in pain modulation, in SIA remains completely unknown. In this study, we investigated the contributions of MORs expressed on glutamatergic (MORGlut) and GABAergic (MORGABA) neurons of the medial prefrontal cortex (mPFC), as well as the functional role and activity of neurons projecting from the mPFC to the periaqueductal gray (PAG) region, in male mice. We achieved this through a combination of hot-plate tests, c-fos staining, and 1 h acute restraint stress exposure tests. The results showed that our acute restraint stress protocol produced mPFC MOR-dependent SIA effects. In particular, MORGABA was found to play a major role in modulating the effects of SIA, whereas MORGlut seemed to be unconnected to the process. We also found that mPFC–PAG projections were efficiently activated and played key roles in the effects of SIA, and their activation was mediated by MORGABA to a large extent. These results indicated that the activation of mPFC MORGABA due to restraint stress was able to activate mPFC–PAG projections in a potential “disinhibition” pathway that produced analgesic effects. These findings provide a potential theoretical basis for pain treatment or drug screening targeting the mPFC.

## 1. Introduction

Stress can induce several bodily changes like emotional sensitization [1,2], formation of aversive memories [1], and increases in synaptic energy metabolism [3], as well as the induction of analgesia [4,5]. Stress triggers decreased nociceptive responses to acute stressor stimuli, such as foot shock, forced swimming, and immobilization. This process is referred to as stress-induced analgesia (SIA) [4,5]. In laboratory settings, various neurotransmitters and neuropeptides—including opioids, cannabinoids, dopamine, oxytocin, and glutamate systems—have been identified to be involved in SIA based on the intensity, duration, and nature of the stressor [4]. In this regard, endogenous opioid systems (EOSs) represent major SIA mechanisms through which to induce SIA via moderate stimuli, such as forced swimming in 32 °C water, acute restraint, or intermittent foot shocks [6,7]. In particular, mu opioid receptors (MORs) are most dominant in EOSs involving SIA [5]. Both pharmacological and genetic evidence confirmed that disturbance of MOR function significantly attenuated SIA effects [6,7,8,9]. In contrast, the anti-nociception effects of EOSs during SIA can be significantly promoted by administrating the MOR agonist morphine [10]. These findings all support the importance of MORs during EOS-mediated SIA.

In terms of the specific mechanism behind MOR-mediated SIA, a number of studies have focused on the descending pain modulatory pathway of the central nervous system (CNS), which comprises the periaqueductal gray (PAG), rostral ventromedial medulla (RVM), and spinal cord [11,12]. Activation of PAG–RVM–spinal cord excitatory projections is potent in terms of inhibiting the ascending transmission of pain information from the spinal cord [11,12]. It has been well studied that MORs are densely expressed in in the inhibitory gamma-aminobutyric acid (GABA)ergic interneurons of the PAG and the RVM [12]. Activation of these MORs has been hypothesized to suppress the activation of these inhibitory interneurons and indirectly activate the PAG–RVM–spinal cord excitatory projections that induce analgesia during SIA [11,12,13]. In addition to the descending pain modulatory pathway, several other forebrain regions that are known to control the processing of pain-related information, such as the cortex, hippocampus, and amygdala, have also been shown to play vital roles in SIA. MORs are extensively distributed in these areas as well [14]; however, it remains unclear whether these MORs are involved in SIA.

The medial prefrontal cortex (mPFC) represents one of the crucial cerebral cortex regions that participate significantly in the transmission and processing of pain responses [15,16,17,18,19,20,21]. It receives terminal neuronal projections from several brain areas that participate in the perception of pain [16], and projects dense fibers to the PAG [17,18,19,20,21]. Both morphological and neurochemical studies have identified that MORs are enriched in the mPFC [14,22]. This evidence supports the notion that the mPFC may represent another target for MORs in MOR-mediated SIA. In addition, MORs are expressed on both glutamatergic (MORGlut) and GABAergic (MORGABA) neurons in the mPFC, and the interaction between MORs on these divergent neuronal types may be involved in the processing of SIA [14,22]. Thus, we hypothesized that MORs expressed in subpopulations of neurons in the mPFC are involved in MOR-dependent SIA.

To test this, we developed three male murine models that were conditionally knocked down for MORs and evaluated the role of mPFC–PAG projections in acute restraint SIA. We also investigated the potential involvement of mPFC GABAergic MORs in the modulation of mPFC–PAG projections using both chemogenetic and morphological techniques. We found that MORGABA, but not MORGlut, plays a major role in modulating acute restraint SIA. Furthermore, mPFC–PAG projections were efficiently activated and played key roles in acute restraint SIA, and their activation was mediated by MORGABA to a large extent. Our present study expounded the role of mPFC MORs expressed in divergent neuronal types on MOR-dependent SIA for the first time. The results verified that besides the descending pain modulatory pathway, cortical areas also play a vital role in MOR-dependent SIA. This would provide a potential theoretical basis for pain treatment targeting the mPFC MORs.

## 2. Results

### 2.1. Acute Restraint Stress Produces mPFC MOR-Dependent SIA

**Figure 1:**
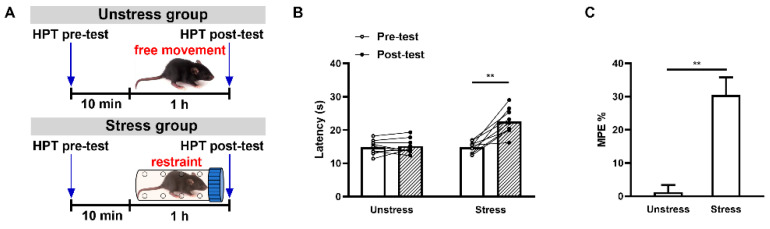
Acute restraint stress-induced analgesia (SIA). (**A**) Diagram of the stress and hot-plate test (HPT) procedures. (**B**) Effects of acute restraint stress on analgesia as assessed via an HPT; *n* = 9 for each group; ** *p* < 0.01. (**C**) The percentage of the maximum possible effect (MPE%) from (**B**), calculated as MPE% = (post-test latency − pre-test latency)/(cut-off latency − pre-test latency) × 100%), the same below; the group data are shown as means ± SEMs; ** *p* < 0.01.

We first determined the acute restraint stress-induced SIA paradigm using the HPT assay. A pain behavioral test was conducted by placing an animal on a 50 °C heated plate and measuring the time until pain-induced reactions (e.g., jumping, paw-flicking, or licking) occurred to determine the animal’s threshold of pain. Previous studies have demonstrated that 1 h restraint tests can trigger steady SIA effects [23,24,25]. After 3 d of handling the mice normally in order to achieve adaptation, we administered 1 h restraint stress tests to the experimental (stress) group but allowed the control (unstressed) group of mice to move freely within the experimental enclosure (Figure 1A). Consistently with the results of previous studies [23,24,25], we found significant analgesic effects in the stress group but only infrequent changes in the unstressed one in terms of both the HPT latency (stress administration: *F*_(1,16)_ = 13.38, *p* = 0.0021; stress duration: *F*_(1,16)_ = 29.76, *p* < 0.0001; interaction: *F*_(1,16)_ = 24.84, *p* = 0.0001; stress pre-test vs. stress post-test, *p* < 0.0001; unstressed pre-test vs. unstressed post-test, *p* = 0.9343; two-way ANOVA and post hoc Sidak multiple-comparisons test; Figure 1B) and percentage of the maximum possible effect (MPE%, calculated as MPE% = (post-test latency − pre-test latency)/(cut-off latency − pre-test latency) × 100%) (*t*_16_ = 5.117, *p* = 0.0001, unpaired Student *t*-test; Figure 1C). These results indicated that 1 h of restraint stress was able to steadily induce an analgesic response.

**Figure 2:**
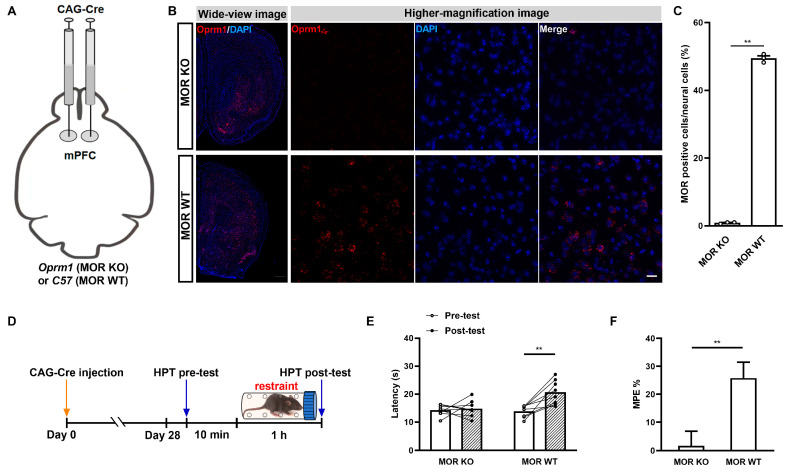
The role of Mu opioid receptors (MORs) in the medial prefrontal cortex (mPFC) in SIA induced through acute restraint. (**A**) Flow diagram of the generation of MOR KO and MOR WT mice. (**B**) (**Left**): Schematic of in situ hybridization for *Oprm1* mRNA in the areas containing the mPFC in MOR KO and MOR WT mice. The *Oprm1* mRNA was stained in red, while the nucleus was stained in blue (DAPI). Scale bar = 500 µm. (**Right**): Higher-magnification images of the fields in the mPFC areas of MOR KO and MOR WT mice. Bar = 20 µm. (**C**) Quantitative analysis of the percentage of neurocyte MORs expressed in the mPFCs of MOR KO and MOR WT mice; *n* = 3 fields containing the median position of the right mPFC (acquired from different *n* = 3 animals) were used per group; MOR-positive cells and total neural cells were counted in each field; the form of data presentation was the MOR-positive cell/neural cell ratio. ** *p* < 0.01. (**D**) Diagram of adeno-associated virus (rAAV) injection, stress, and HPT procedures. (**E**) Contributions of mPFC MORs to SIA induced through acute restraint as assessed via an HPT; *n* = 8 for each group; ** *p* < 0.01. (**F**) Equated MPE% from the groups in (**E**), and data are shown as means ± SEMs; ** *p* < 0.01.

Next, we explored whether mPFC MORs were involved in acute restraint SIA. To achieve this, rAAV2/9-CAG-CRE-WPRE-hGH-pA were bilaterally injected into the mPFCs of *Oprm1*^flox^ or *C57 BL/6J* mice of the same age to generate PFC MOR knockout (MOR KO) mice or MOR wild-type (MOR WT) control mice, respectively (Figure 2A). Using fluorescence in situ hybridization with RNAscope technology, we tested the knockdown efficiency in the MOR KO mice. We found that almost all of the MORs were undetectable in the mPFCs of MOR KO mice, in contrast to the MOR WT mice, where dense MORs remained present in the mPFC (Figure 2B). Further statistical results for the morphology indicated a significant reduction in MORs in the mPFCs of the MOR KO mice (*p* < 0.0001; Student *t*-test; Figure 2C). These results indicated a powerful knockdown efficiency of cre-enzyme-mounted rAAVs. Therefore, we proceeded to explore the related changes in the effects of SIA during the restraint stress test in the mPFC MOR KO mice (Figure 2D). We found that the effects of SIA were significantly inhibited in the mPFC MOR KO mice, which was in contrast with what took place in the MOR WT mice (rAAV administration: *F*_(1,14)_ = 6.485, *p* = 0.0233; stress duration: *F*_(1,14)_ = 12.81, *p* = 0.0030; interaction: *F*_(1,14)_ = 8.948, *p* = 0.0097; MOR KO pre-test vs. MOR KO post-test, *p* = 0.0008; MOR WT pre-test vs. MOR WT post-test, *p* = 0.9002; two-way ANOVA post hoc Sidak multiple-comparison test; Figure 2E). The MPE% further showed evident differences in the effects of SIA between the MOR KO and MOR WT mice (*t*_14_ = 3.157, *p* = 0.0070, unpaired Student *t*-test; Figure 2F). These results indicated the crucial role of mPFC MORs in the modulation of acute restraint SIA.

### 2.2. GABAergic but Not Glutamatergic MORs in the mPFC Modulate MOR-Dependent SIA

It has been previously demonstrated that MORs are expressed on both glutamatergic and GABAergic neurons and that both are involved in pain modulation [14,22]. To address the distinct effects of mPFC MORGlut and MORGABA on analgesia under acute restraint stress conditions, we knocked down MORGlut or MORGABA using neural-specific cre-enzyme-mounted rAAVs to create mPFC MORGlut-conditioned KO (MORGlut cKO) or MORGABA-conditioned KO (MORGABA cKO) mice.

**Figure 3:**
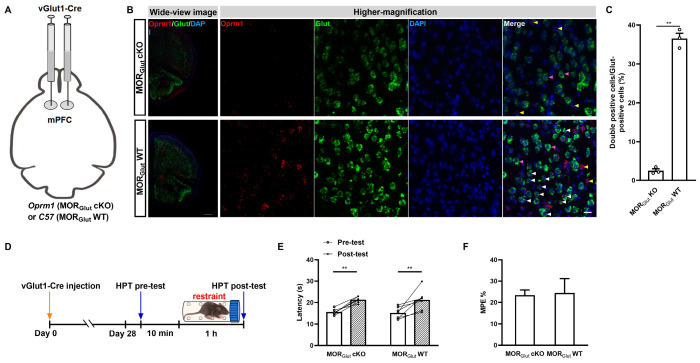
MORGlut in the mPFC plays a marginal part in the modulation of SIA induced by acute restraint. (**A**) Diagram of the generation of MORGlut cKO and MORGlut WT mice. (**B**) (**Left**): Schematic of in situ hybridization for *Oprm1* mRNA in the areas containing mPFC in MORGlut cKO and MORGlut WT mice. The *Oprm1* mRNA was stained in red, *vGlut1* mRNA was stained in green, and the nuclei were stained in blue (DAPI). Bar = 500 µm. (**Right**): Higher-magnification images of the fields in the mPFC areas in MORGlut cKO and MORGlut WT mice. The white arrowhead indicates a double-labeled cell with *Oprm1* mRNA and *vGlut1* mRNA, the yellow arrowheads represent *Oprm1* mRNA localization in *vGlut1*-negative cells, and the purple arrowheads represent *vGlut1*-positive cells without Oprm1 mRNA. Bar = 20 µm. (**C**) Quantitative analysis of the percentage of MORGlut expressed in the mPFCs of MORGlut KO and MORGlut WT mice; *n* = 3 fields containing the median position of the right mPFC (acquired from different *n* = 3 animals) were used per group; the double-positive cells and total Glut-positive cells were counted in each field; the form of data presentation was the double-positive cell/Glut-positive cell ratio; ** *p* < 0.01. (**D**) Flow diagram of the rAAV injection, stress, and HPT procedures. (**E**) Rare contributions of mPFC MORGlut to SIA induced by acute restraint as assessed via an HPT; *n* = 7 for each group; ** *p* < 0.01. (**F**) Equated MPE% from the groups in (**E**); data are shown as means ± SEMs.

First, we explored the role of mPFC MORGlut in acute restraint SIA. We injected rAAV2/9-VGLUT1-CRE-WPRE-hGH-pA and neural-specific rAAVs targeting glutamatergic neurons into the bilateral mPFCs of *Oprm1*^flox^ mice or *C57 BL/6J* mice of the same age to generate mPFC MORGlut cKO or MORGlut WT control mice (Figure 3A). Fluorescence in situ hybridization using an RNAscope indicated that the MORGlut levels were significantly decreased in the MORGlut cKO mice compared with the MORGlut WT mice (Figure 3B). A significant reduction in the MORGlut cKO mice was evident in the statistical results of our morphological assays (*p* < 0.0001; Student *t*-test; Figure 3C). These indicated the strong knockdown efficiency of MORGlut in MORGlut cKO mice. Thus, we further proceeded to assess the impacts of MORGlut cKO or MORGlut WT on acute restraint SIA (Figure 3D). We found that both MORGlut cKO and MORGlut WT mice exhibited prominent SIA after acute restraint stress in the HPT (rAAV administration: *F*_(1,12)_ = 0.02978, *p* = 0.8659; stress duration: *F*_(1,12)_ = 43.51, *p* < 0.0001; interaction: *F*_(1,12)_ = 0.03510, *p* = 0.8545; MORGlut cKO pre-test vs. MORGlut cKO post-test, *p* = 0.0014; MORGlut WT pre-test vs. MORGlut WT post-test, *p* = 0.0009; MORGlut WT post-test vs. MORGlut cKO post-test, *p* = 0.9994; two-way ANOVA and post hoc Sidak multiple-comparison test; Figure 3E). The MPE% showed no differences in SIA between MORGlut cKO and MORGlut WT mice (*t*_12_ = 0.1407, *p* = 0.8904, unpaired Student *t*-test, Figure 3F).

**Figure 4:**
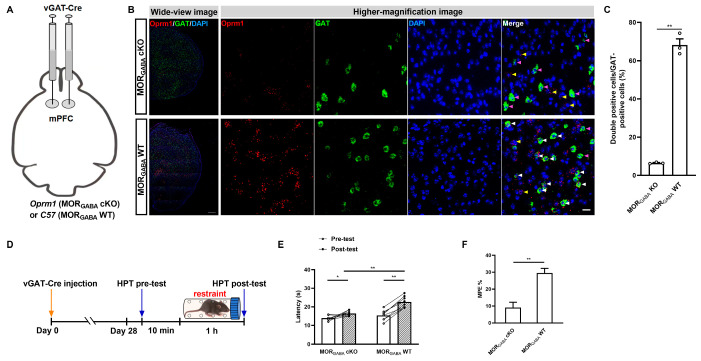
Contributions of mPFC MORGABA to SIA induced by acute restraint. (**A**) Diagram detailing the generation of MORGABA cKO and MORGABA WT mice. (**B**) (**Left**): Schematic of in situ hybridization for *Oprm1* mRNA in the areas containing mPFC in MORGABA cKO and MORGABA WT mice. The *Oprm1* mRNA was stained in red, *vGAT* mRNA was stained in green, and nuclei were stained in blue (DAPI). Bar = 500 µm. (**Right**): Higher-magnification images of the fields in the mPFC areas in MORGABA cKO and MORGABA WT mice. The white arrowhead indicates a double-labeled cell with *Oprm1* mRNA and *vGAT* mRNA, the yellow arrowheads represent *Oprm1* mRNA localization in *vGAT*-negative cells, and the purple arrowheads represent *vGAT*-positive cells without Oprm1 mRNA. Bar = 20 µm. (**C**) Quantitative analysis of the percentage of MORGABA expressed in the mPFCs of MORGABA KO and MORGABA WT mice; *n* = 3 fields containing the median position of the right mPFC (acquired from different *n* = 3 animals) were used per group; the double-positive cells and total GAT-positive were counted in each field; the form of data presentation was the double-positive cell/GAT-positive cell ratio; ** *p* < 0.01. (**D**) Flow diagram of the rAAV injection, stress, and HPT procedures. (**E**) Contributions of mPFC MORGABA to SIA induced by acute restraint as assessed via an HPT; *n* = 8 for each group; * *p* < 0.05; ** *p* < 0.01. (**F**) Equated MPE% from the groups in (**E**), and the data are shown as means ± SEMs; ** *p* < 0.01.

Next, we determined the role of mPFC MORGABA in acute restraint SIA. The specific GABAergic neuron-targeting rAAV2/9-VGAT-CRE-WPRE-hGH-pA was bilaterally injected into the mPFCs of *Oprm1*^flox^ mice or *C57 BL/6J* mice of the same age to generate mPFC MORGABA cKO and MORGABA WT control mice (Figure 4A). The morphological results showed that MORGABA receptors were knocked down in the MORGABA cKO mice, unlike in the MORGABA WT mice (Figure 4B). The statistical results showed that the MORGABA levels were significantly reduced in the MORGABA cKO mice (*p* < 0.0001; Student *t*-test; Figure 4C). This indicated a powerful knockdown efficiency in the MORGABA mice. We then detected the effects of acute restraint SIA on both the MORGABA cKO and MORGABA WT mice under exposure to acute restraint stress (Figure 4D). The results indicated that, although the HPT latencies of both groups of mice showed significant increases after the stress was administered, the increased lag between stress exposure and response seen in the MORGABA cKO mice indicated significant inhibition compared with the responses of the MORGABA WT mice (rAAV administration: *F*_(1,14)_ = 14.10, *p* = 0.0021; stress duration: *F*_(1,14)_ = 83.15, *p* < 0.0001; interaction: *F*_(1,14)_ = 20.01, *p* = 0.0005; MORGABA cKO pre-test vs. MORGABA cKO post-test, *p* = 0.0108; MORGABA WT pre-test vs. MORGABA WT post-test, *p* < 0.0001; MORGABA WT post-test vs. MORGABA cKO post-test, *p* < 0.0001; two-way ANOVA and post hoc Sidak multiple-comparison test; Figure 4E). The MPE% further revealed this difference between the groups (*t*_14_ = 4.915, *p* = 0.0002, unpaired Student *t*-test; Figure 4F). These results indicated that mPFC MORGABA receptors partially participate in the modulation of SIA under exposure to acute restraint stress.

### 2.3. GABAergic MORs in the mPFC Modulate the Activity of mPFC–PAG Projections under the MOR-Dependent SIA Paradigm

**Figure 5:**
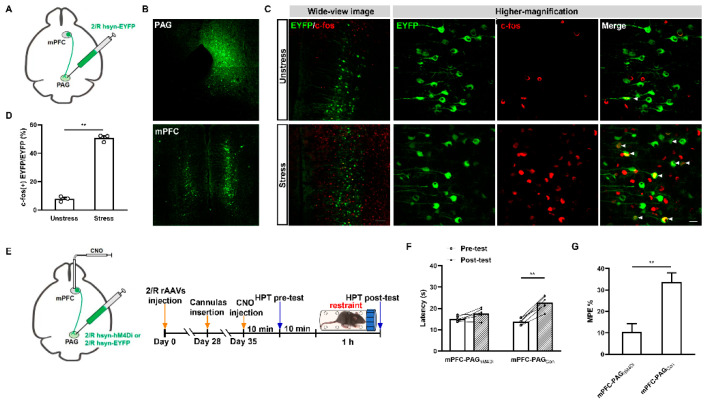
mPFC–periaqueductal gray (PAG) projections are significant in the process of inducing SIA through acute restraint. (**A**) Diagram showing the labeling of mPFC–PAG projections. (**B**) Typical morphology of labeled mPFC–PAG projections. Bar = 200 µm. (**C**) (**Left**): Representative images showing c-fos expression in mPFC–PAG projections in our stressed and unstressed groups of mice. Bar = 100 µm. (**Right**): Higher-magnification images of the fields from the left. The white arrowhead indicates a double-labeled cell with labeled mPFC–PAG projections and c-fos. Bar = 20 µm. (**D**) Quantitative analysis of the percentage of c-fos positive-labeled mPFC–PAG projections from the mPFCs of mice in the stressed and unstressed groups; *n* = 3 fields containing the median position of the right mPFC (acquired from different *n* = 3 animals) were used per group; the c-fos-positive EYFP cells and total EYFP cells were counted in each field; the form of data presentation was the c-fos-positive EYFP cell/EYFP cell ratio. ** *p* < 0.01. (**E**) Diagram detailing the mounting of an inhibitory chemogenetical module in mPFC–PAG projections and a flow diagram of rAAV injection, cannula insertion, CNO injection, stress, and HPT procedures. (**F**) The influence of the chemogenetic inhibition of mPFC–PAG projections on SIA induced by acute restraint as assessed via an HPT; *n* = 6 for each group; ** *p* < 0.01. (**G**) Equated MPE% from the groups in (**F**), and data are shown as means ± SEM; ** *p* < 0.01.

mPFC–PAG projections are essential for pain reactions [19]. To test whether these projections are involved in acute restraint SIA, rAAV2/R-hsyn-EYFP-WPRE-hGH-pA (retrograde nerve-tracing rAAVs) were injected into the PAG structures of our mice to trace the mPFC–PAG projections (Figure 5A). This allowed the clear labeling of the projections, as shown in Figure 5B. Thus, we were able to detect the activity levels of mPFC–PAG projections under exposure to acute restraint stress using a combination of retrograde tracing with rAAVs and c-fos staining. We found that many of the mPFC–PAG projections exhibited positive c-fos characteristics after 1 h of acute restraint stress, whereas only minute of c-fos detection was present in the unstressed control mice (Figure 5C). This was further confirmed via statistical analysis of the results (*t*_4_ = 23.22, *p* < 0.0001, unpaired Student *t*-test; Figure 5D). This indicated that mPFC–PAG projections were significantly activated under conditions of acute restraint stress exposure. To further investigate whether the activation of mPFC–PAG projections was necessary to achieve the effects of SIA with exposure to acute restraint stress, rAAV2/R-hsyn-hM4Di-EYFP-WPRE-hGH-pA—retrograde-tracing rAAVs that functionally inhibit the activity of these projections when administered in conjunction with CNO—were injected into the PAGs of *C57 BL/6J* mice according to a previously described protocol [19]. The control mice received only rAAV2/R-hsyn-EYFP-WPRE-hGH-pA. After rAAV expression was allowed to take place over 28 days and cannulas were inserted for 7 days in the mPFCs, we administered CNO into the mPFCs and proceeded with pre- and post-acute restraint stress testing using an HPT (Figure 5E). We found that, unlike in the control mice, CNO significantly reduced the activity levels of mPFC–PAG projections, lessening the effects of SIA, in the experimental mice. This was evident in terms of both latency in the HPT (rAAV administration: *F*_(1,10)_ = 3.235, *p* = 0.1023; stress duration: *F*_(1,10)_ = 61.36, *p* < 0.0001; interaction: *F*_(1,10)_ = 17.80, *p* = 0.0018; mPFC-PAG_hM4Di_ pre-test vs. mPFC-PAG_hM4Di_ post-test, *p* = 0.0564; mPFC-PAG_Con_ pre-test vs. mPFC-PAG_Con_, *p* < 0.0001; two-way ANOVA and post hoc Sidak multiple-comparison test; Figure 5F) and MPE% (*t*_10_ = 4.058, *p* = 0.0023, unpaired Student *t*-test; Figure 5G). These results indicated that mPFC–PAG projections played a vital role in acute restraint SIA.

**Figure 6:**
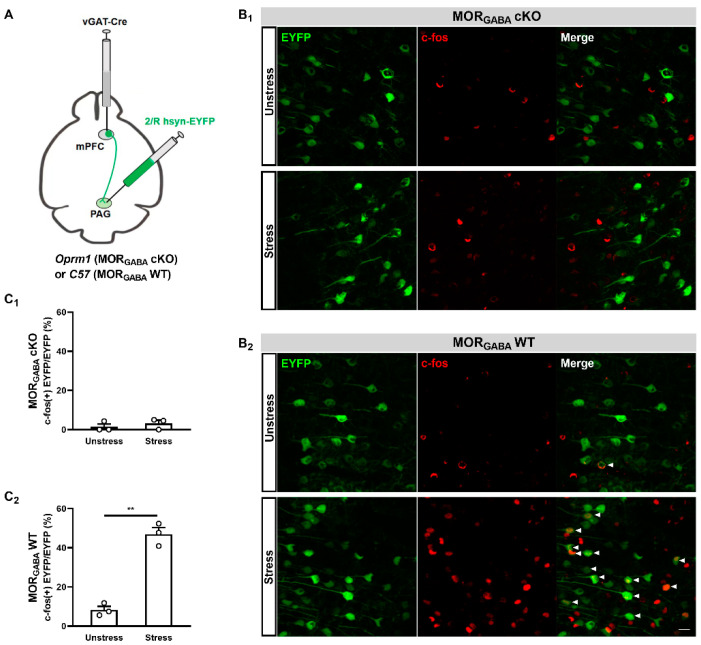
MORGABA modulated the activity of mPFC–PAG projections during MOR-dependent SIA. (**A**) Diagram detailing the labeling of mPFC–PAG projections in MORGABA cKO and MORGABA WT mice. (**B**) Representative higher-magnification images showing c-fos expression in mPFC–PAG projections for MORGABA cKO (**B_1_**) and MORGABA WT (**B_2_**) mice under acute restraint stress or unstressed conditions. Bar = 20 µm; *n* = 3 fields containing the median position of the right mPFC (acquired from different *n* = 3 animals) were used per group; The white arrowheads represent co-labeling of EYFP and c-fos, the c-fos-positive EYFP cells and total EYFP cells were counted in each field; the form of data presentation was the c-fos-positive EYFP cell/EYFP cell ratio; ** *p* < 0.01. (**C**) Quantitative analysis of the percentage of c-fos(+)-labeled mPFC–PAG projections in the mPFCs of MORGABA cKO (**C_1_**) or MORGABA WT (**C_2_**) mice under acute restraint stress or unstressed conditions; 3 fields were quantified from 3 animals; ** *p* < 0.01.

Next, we explored whether mPFC MORGABA receptors were involved in the modulation of mPFC–PAG projections during SIA using retrograde tracing of the mPFC–PAG projections in both MORGABA WT and MORGABA cKO mice (Figure 6A). After allowing rAAVs to be expressed in the mPFCs, we detected neural activity levels in the mPFC–PAG projections with or without acute restraint stress by using c-fos staining. In agreement with our previous results, the mPFC–PAG projections of the MORGABA WT mice showed significant enhancements of positive c-fos characteristics under exposure to acute restraint stress, which was not observed in the unstressed mice (Figure 6B_2_). However, the same tendency was not seen in the MORGABA cKO mice. Several positive c-fos characteristics were observed during both the pre- and post-acute restraint stress conditions (Figure 6B_1_). Our statistical results also exhibited rare changes (*t*_4_ = 0.8036, *p* = 0.4667, unpaired Student *t*-test; Figure 6C_1_), whereas a significant increase in c-fos-positive characteristics (*t*_4_ = 9.999, *p* = 0.0006, unpaired Student *t*-test; Figure 6C_2_) was found in the MORGABA cKO and MORGABA WT mice, respectively. These results indicated that mPFC MORGABA was potentially involved in the modulation of the mPFC–PAG projection activity levels under the MOR-dependent SIA paradigm, suggesting that the activation of mPFC MORGABA may activate mPFC–PAG projections to produce SIA with exposure to acute restraint stress.

## 3. Discussion

This study clarified the functional role of mPFC MORs in terms of the modulation of acute restraint stress-induced MOR-dependent SIA in male mice. As hypothesized, GABAergic but not glutamatergic MORs played the predominant role in analgesic effects. Additionally, mPFC–PAG projections were involved in this form of SIA and were mediated by GABAergic MORs to some extent. Restraint stress activated mPFC GABAergic MORs to relieve the inhibitory effects of mPFC GABA neurons on mPFC–PAG projections, resulting in SIA.

Our present study confirmed the contribution of mPFC MORs, mainly GABAergic MORs, on 1 h restraint stress-induced SIA in male mice. Two previous studies have reported that 30 min of restraint stress in C57/BL6J mice, and 1 h of restraint stress in rats, can induce MOR-contributed SIA [24,26]. The stress duration and intensity of the present study is higher than in previous studies [24,26], and a remarkable MOR-contributed SIA effect was also exhibited (Figure 2). This result suggested that the MOR-contributed SIA can be present in a relatively wide range of restraint stress durations, and mPFC MORs are involved in this process.

With the progression of evolution, the challenges that animals face have become increasingly complex. Accordingly, stress has become a prevalent aspect of animal life that necessitates timely responses. SIA represents a typical pain-reducing response that enables animals to focus more effectively on stressful stimuli, thereby better dealing with stress and avoiding potential danger [4]. SIA is a systematic process that necessitates collaborative mediation between several pain-related brain areas. Previous studies have demonstrated the role of the EOS in the descending pain inhibitory pathway during SIA [4,27,28]; however, brain areas outside of the descending pain inhibitory pathway have rarely been discussed in the literature. This study elucidated the EOS-mediating mechanism of the mPFC in terms of modulating SIA induced by acute restraint. We found that MOR deletion in the mPFC largely abolished the effects of SIA. As there were no statistically significant differences in the basal pain threshold levels between our MOR KO and MOR WT control mice, the MOR KO mice exhibited a notable deficiency in SIA after stress was administered, which was evident in terms of both HPT latencies and calculated MPE% values (Figure 2). The mPFC not only receives pain information from the spinal cord but also sends projections targeting the descending pain inhibitory pathway [16]. One human study involving millisecond-long electroencephalography reported that an increase in gamma and theta power in the mPFC is relevant to enhanced pain perception [21]. Moreover, it has been clarified that noxious stimuli can increase the firing rates of mPFC neurons to compete with pain neurons [29], whereas a significant decrease in mPFC neuronal activity was observed in both patients with migraines and animal models of chronic pain [29,30]. Optogenetically increasing the activity of mPFC neurons scales up prefrontal outputs to inhibit pain responses [29]. This study further explored the preliminary functional role of the mPFC in acute restraint SIA, which may be helpful for understanding the cortical mechanism underlying SIA in the CNS.

It has been reported that several neurotransmitters and neuropeptides—including opioids, cannabinoids, monoamines, GABA, and glutamate systems—are involved in SIA [4,31]. Among these, the EOS is known as a critical constituent of the SIA mechanism, particularly when SIA is induced by acute or transient stimuli [4,5]. This study used a 1 h acute restraint stress, a classical MOR-dependent SIA paradigm, to demonstrate the roles of MORs expressed in different populations of mPFC neurons involved in the SIA process [23]. We found that mPFC MORGABA but not MORGlut significantly contributed to SIA modulation (Figure 3 and Figure 4). Recent studies have identified functional MORs expressed in both glutamatergic and GABAergic neurons in several pain-related brain areas, such as the hippocampus, VTA, PAG, RVM, and cortex [14]. In the physiology of pain modulation, MORGlut mainly participates in exogenous opioid analgesia (e.g., morphine-induced analgesia), primarily targeting the spinal cord [14,22]. In contrast, MORGABA is more efficient for inducing endogenous opioid analgesia [22]. This study found that a MORGABA cKO phenotype in the mPFC significantly weakened the effects of SIA (Figure 3). This is consistent with the results of certain previous studies, which showed that the optogenetic or chemogenetic inhibition of mPFC GABAergic neurons increased analgesic effects [19,32]. MORs are typical inhibitory G protein-coupled receptors (GPCRs) that are expressed in the CNS [33,34]. The activation of MORs expressed on neurons can interrupt influxes of sodium and calcium, thereby weakening neuronal activation [33,35]. Thus, during the SIA process, activation of mPFC MORGABA seems to inhibit GABAergic neurons, indirectly activating excitatory projecting neurons originating from the mPFC in a process known as disinhibition [11,13]. As a result, the descending pain inhibitory pathway is activated through neural transmission from mPFC–PAG projections, leading to analgesia. These results illustrate the vital role of mPFC MORGABA in MOR-dependent SIA. In contrast, we did not identify any effects of mPFC MORGlut on SIA induced by acute restraint. The HPT showed insignificant differences between mPFC MORGlut cKO and control mice (Figure 4). A similar previous study clarified that endogenous activation of vGlut2-positive MORGlut minimally contributed to pain modulation [14]. Furthermore, vGlut1-positive MORGlut tended to inadequately contribute to analgesic effects in an animal model that used 3 min of forced swimming in warm (32 °C) water to induce SIA [22]. In addition to pain modulation, the mPFC participates in a number of other brain-related functions, such as decision making, learning, memorizing, and emotional responses, such as anxiety and aversion [36,37,38]. Thus, the expression of MORs on mPFC glutamatergic neurons may be involved in the modulation of these cognitive or emotional functions. Notably, the effective reduction in SIA in our MORGABA cKO mice did not completely reach the level seen in the MOR KO mice (Figure 3). Therefore, it seems that there may be other components that are also involved in the modulation of SIA induced by acute restraint in the mPFC. Indeed, recent studies have also identified functional MORs expressed in glial cells, such as astrocytes. In this regard, murine astrocytic MORs have been confirmed to be involved in the modulation of synaptic activity through the calcium-signaling-dependent release of gliotransmitters or TREK-1-containing two-pore potassium (K2P) channels, controlling the release of glutamate and, thus, participating in several higher neural activities, such as conditioned place preference [39,40]. The mPFC contains a number of astrocytes that are involved in the modulation of chronic pain [41,42]. Thus, the activation of MORs expressed in these cells may represent another mechanism underlying the modulation of MOR-dependent SIA. Further experiments are warranted to confirm this notion.

This study further clarified the involvement of mPFC–PAG projections in SIA induced by acute restraint and the potential modulatory role of mPFC MORGABA in these projections. Our morphological results indicated that many of the mPFC–PAG projections were activated under restraint stress exposure and that the chemogenetic inhibition of these projections significantly reversed the restraint-induced effects of SIA. These results suggest a crucial role of mPFC–PAG projections in the modulation of SIA induced by acute restraint (Figure 5). As the origin of the descending pain inhibitory pathway, the PAG participates significantly in pain modulation. Electrical stimulation of the PAG region was shown to suppress responsiveness to various noxious stimuli—including electric shock, tissue-destructive pinches, and radiant heat applied to the tail [43]—whereas lesions of the PAG disrupted antinociceptive signaling in a fear-conditioned rat model of analgesia [44]. In a study involving fiber photometry recording, the activity levels of PAG neurons that projected to the RVM were found to be significantly increased in a MOR-dependent manner under exposure to stress induced by forced swimming [22]. These studies support the crucial role of the PAG in modulating SIA. The mPFC is one of the main cortical areas that extends excitatory projections into the PAG. A recent study reported that mPFC–PAG excitatory projections have direct innervation to PAG–RVM vGlut2-positive projections [19]. Optogenetic activation of these projections was shown to attenuate common peroneal-nerve-ligation-induced mechanical hyperalgesia, whereas the inhibition of neuronal activity in these projections led to the aggravation of mechanical hyperalgesia [19]. Thus, the activation of mPFC–PAG projections may further activate the descending pain inhibitory pathway, thereby inducing analgesia. The results of this study were consistent with those of previous ones in this regard. The effects of SIA due to acute restraint stress were accompanied by the activation of mPFC–PAG projections, as observed in both behavioral tests and via c-fos staining, clarifying the neural conditions present during the activation of the descending pain inhibitory pathway when it produces analgesia. Notably, we found that the MORGABA cKO phenotype in the mPFC suppressed the activation of mPFC–PAG projections under exposure to acute restraint stress. Immunofluorescence indicated a significant decrease in c-fos expression in MORGABA cKO mPFC–PAG projections following restraint-induced stress (Figure 6). These results suggest that the activation of mPFC–PAG projections may be modulated by mPFC MORGABA during the SIA process. The disinhibition of mPFC–PAG projections triggered by mPFC MORGABA activation may represent one of the mechanisms underlying SIA induced by acute restraint. It is important to note, however, that the results of this study apply only to male mice. It has been reported that female mice exhibit non-negligible differences in terms of MOR-dependent pain modulation when compared with males [45]. Thus, more studies on female mice are warranted to better understand the modulatory role of mPFC MORs during SIA. In addition, there is also a certain deficiency in the morphological analysis of our present study. Our methodological limitations of the quantitative analysis are that only three animals were used and only three fields per group were analyzed. Although the animal selection was random, it is not sufficient to say that the same is true in each individual of group. More work should be repeated in the future to ensure the authenticity of the morphological analysis in our present study.

This study illustrated the involvement of mPFC MORGABA in SIA induced by acute restraint in male mice. The activation of mPFC MORGABA through stress was able to activate mPFC–PAG projections in a potential disinhibition pathway, thereby producing analgesic effects. Our findings provide a potential theoretical basis for pain treatment or drug screening targeting the mPFC.

## 4. Materials and Methods

### 4.1. Animals

All animals used in this study were used according to the guidelines of the Chinese Animal Protection Commission, and the study was approved by the Animal Protection Committee of Shaanxi Normal University. A total of 62 *C57 BL/6J* (8–12-week-old) and 26 *Oprm1*^flox^ (8–12-week-old) male mice were used in the study, and they were provided by the Experimental Animal Center of Shaanxi Normal University MOE Key Laboratory of Modern Teaching Technology. The strategy of mouse use is listed as follows: 18 *C57 BL/6J* mice were used to establish the acute restraint stress-induced SIA paradigm; 23 *C57 BL/6J* and *23 Oprm1*^flox^ mice were used to detect the role of mPFC MORs, MORGlut, and MORGABA on the modulation of SIA; 18 *C57 BL/6J* mice were used to detect the role of mPFC–PAG projections on the modulation of SIA; 3 *C57 BL/6J* and *3 Oprm1*^flox^ mice were used to detect the contributions of mPFC GABAergic MORs to the modulation of the activity of mPFC–PAG projections under the SIA paradigm. All of the animals were housed in groups of three to five in individually ventilated cages that were maintained at 21 ± 1 °C and 55 ± 5% relative humidity under a 12 h light/dark cycle. All behavioral experiments were conducted between 9:00 and 11:00 A.M. The animals were habituated for 5 d to acclimate them to the environment and apparatus before the experiments began.

### 4.2. Restraint Stress

The restraint stress tests were performed according to previously described protocols [23,24,25]. The mice were placed into cylindrical restraint tubes that were 25 mm in diameter (inner diameter) and 60 mm long (straight part) with 20 mm long fixed valves (containing ventilation holes to facilitate normal respiration). Each tube was placed in an individually ventilated chamber for 1 h to induce stress. The unstressed control mice were also placed inside the ventilated chambers but were not restrained for 1 h.

### 4.3. Pain Behavioral Test

A hot-plate test (HPT) was used to assay anti-nociception levels in the animals according to a previously described protocol [26]. The mice were placed on a 50 °C hot plate, and the time until pain-induced reactions (e.g., jumping, paw-flicking, or licking) occurred was measured using a stopwatch. For each mouse, two latency sessions of HPTs—comprising 10 min before the stress (i.e., the pre-test) and 10 min immediately following the stress (i.e., the post-test)—were conducted. Each session comprised two tests, and the average lag between placement on the plate and the pain-induced response was used as the final result. The pre-stress times of the mice were controlled at ~10–15 s in order to reduce variability between individuals. The cut-off time was set to 45 s in order to avoid foot tissue damage. Aside from the latencies, the HPT results were also expressed as the percentage of the maximum possible effect (MPE%), which was calculated as follows: MPE% = (post-test latency − pre-test latency)/(cut-off latency − pre-test latency) × 100%.

### 4.4. Surgery and Adeno-Associated Virus Injection

The animals were anesthetized with 4% isoflurane and fixed in place using a stereotaxic brain apparatus. A 1% isoflurane inhalant was continually delivered to the nasopharynx of each mouse to maintain anesthesia during surgery. After exposing the skull, the following adeno-associated viruses (rAAVs) were injected into designated brain areas: rAAV2/9-CAG-CRE-WPRE-hGH-pA (150 nL/injection, 5.28 × 10^12^ vg/mL; brain VTA), rAAV2/9-VGLUT1-CRE-WPRE-hGH-pA (150 nL/injection, 5.83 × 10^12^ vg/mL; brain VTA), or rAAV2/9-VGAT-CRE-WPRE-hGH-pA (300 nL/injection, 2.61 × 10^12^ vg/mL; brain VTA) was injected into the bilateral mPFC (anteroposterior [AP]: 1.80 mm; mediolateral [ML]: ±0.2 mm; dorsoventral [DV]: −2.40 mm) to create MOR KO, MORGlut cKO, or MORGABA cKO in the mPFCs of *Oprm1*^flox^ mice. These rAAVs were also injected into the bilateral mPFCs of *C57 BL/6J* mice of the same ages as controls (MOR WT, MORGlut WT, or MORGABA WT). In addition, rAAV2/R-hsyn-hM4Di-EYFP-WPRE-hGH-pA (350 nL/injection, 5.72 × 10^12^ vg/mL; brain VTA) or rAAV2/R-hsyn-EYFP-WPRE-hGH-pA (350 nL/injection, 5.80 × 10^12^ vg/mL; brain VTA) was injected into the PAG (AP: −4.80 mm; ML: 0.35 mm; DV: −2.90 mm) to chemogenetically inhibit mPFC–PAG activation (combined with the use of CNO [10 µM, 0.2 µL/injection; brain VTA] administered locally to the mPFC) or morphologically explore the activating effects of mPFC–PAG projections via c-fos staining. After adeno-associated virus injection, dissected head skins were carefully sutured using absorbable sutures and a diclofenac sodium gel was then smeared near the wounds. All animals were allowed to recover for at least 4 weeks before subsequent experiments.

### 4.5. Fluorescence In Situ Hybridization Using an RNAscope

Fluorescence in situ hybridization was used to detect the deletion of MORs in mPFC MOR KO, MORGlut cKO, and MORGABA cKO mutant mice according to previously described protocols [22,46]. Briefly, the mice were deeply anesthetized with pentobarbital sodium (50 mg/kg mouse body weight, intraperitoneal injection) and perfused with 0.9% saline for 5 min. Their brains were then quickly removed and embedded in OCT (catalog no.: Tissue-Tek 4583; Sakura Finetek, Torrance, CA, USA) at −22 °C. Fresh frozen sections (15 µm) containing the mPFC region were cut using a CM1950 freezing microtome (Leica Biosystems, Nussloch, Germany). The sections were fixed in 4% PFA for 15 min at 4 °C and then dehydrated using three grades of ethanol (50%, 75%, and 100%) at room temperature for 5 min each. The sections were incubated with hydrogen peroxide for 15 min and protease IV for 20 min. The probes for vglut1 (catalog no.: 416631-C1), *Oprm1* (catalog no.: 544731-C2), and vgat (catalog no.: 424548-C3) were provided by Advanced Cell Diagnostics (ACD, Newark, CA, USA) and conjugated to Atto 520, Atto 570, and Atto 650, respectively. The procedure for in situ hybridization was implemented according to the manufacturer’s protocol for an RNAscope Multiplex Fluorescent Reagent Kit (catalog no.: 323100; ACD) using a HybEZTM oven (ACD). Finally, the sections were mounted with anti-fade mounting medium containing DAPI. Images were captured using a fluorescence microscope (Zeiss, Oberkochen, Germany). In the counting statistics of MOR KO mutant mice, 1 section was taken from each animal and the same 600 µm × 500 µm size of 1 rectangle image containing the median position of the right mPFC was cut out to manually count the MOR-positive cells and neural cells. The form of data presentation was the MOR-positive cell/neural cell ratio (%). The data generated during this section are detailed in the Supplementary Files (Table S1). In the counting statistics of MORGlut cKO or MORGABA cKO mutant mice, the section and image acquiring was the same as above. The double-positive cells and total Glut-positive (for MORGlut cKO mutant mice) or total GAT-positive (for MORGABA cKO mutant mice) cells were manually counted. The form of data presentation was the double-positive cell/Glut-positive or GAT-positive cell ratio (%). The data generated during this section are detailed in Supplementary Files (Tables S2 and S3).

### 4.6. C-Fos Immunofluorescence

The c-fos immunofluorescence method was used to assess the activating effects of mPFC–PAG projections in *C57 BL/6J* or MORGABA cKO mice before and 1 h following restraint stress. The mice were deeply anesthetized using sodium pentobarbital and successively perfused with 0.9% saline and 4% paraformaldehyde (dissolved in 0.1 M phosphate-buffered saline [PBS]). Whole brains were removed, post-fixed for 24 h, and then immersed in 30% sucrose (dissolved in 0.1 M PBS) for 48 h at 4 °C. The brains were then removed and embedded in OCT, and frozen sections (15 µm) containing the mPFC were cut. For immunofluorescence, the sections were acclimated to room temperature and incubated in PBS containing 10% non-immune donkey serum and 0.5% Triton X-100 for 60 min. They were then incubated overnight at 4 °C with rabbit anti-c-fos (1:500; #2250; Cell Signaling Technology, Danvers, MA, USA). The following day, the sections were washed three times with 0.1 M PBS and incubated with Alexa Fluor 594-conjugated donkey anti-rabbit (1:1000; ab150076; Abcam, Boston, MA, USA) in a light-protected environment for 2 h. The sections were then mounted using an anti-fade mounting medium, and fluorescence imaging was conducted using a fluorescence microscope. In counting statistics, 1 section was taken from each animal, and the same 600 µm × 500 µm size of 1 rectangle image containing the median position of the right mPFC was cut out to manually count the c-fos-positive EYFP cells and total EYFP cells. The form of data presentation was the c-fos-positive EYFP cell/EYFP cell ratio (%). The data generated during this section are detailed in the Supplementary Files (Tables S4 and S5).

### 4.7. Intra-mPFC Injection

Intra-mPFC injections were used to locally deliver CNO into the mPFCs to specifically inhibit the activation of mPFC–PAG projections. Following complete expression of rAAV2/R-hsyn-hM4Di-EYFP-WPRE-hGH-pA on the mPFCs, cannulas were bilaterally inserted into the murine mPFCs. In short, mice were anesthetized with 4% isoflurane, and the heads were stabilized using a stereotaxic brain apparatus, and a 1% isoflurane inhalant was continually delivered to the nasopharynx of each mouse to maintain anesthesia. After skull exposure, the cannulas were inserted into the mPFCs, and the upper-half parts were secured to the skull by using animal glass cements. The cannulas were pre-covered by protective caps before intra-mPFC injections to prevent cannulas clogging. The postoperative management was identical to the surgery of rAAVs injection.

The intra-mPFC injections were carried out after 7 d of recovery. In short, CNO (10 µM, 0.2 µL/injection; brain VTA, China) was aspirated into 0.5 µL needle-tipped micro-syringes (controlled using a micro-infusion pump). Micro-syringes were connected with cannulas by using a plastic hose. Then, CNO was delivered into the cannulas through the plastic hose at a delivery rate of 0.1 µL/min. To ensure complete drug absorption, the needles were left in place for at least 3 min before removal and subsequently rinsed with ultrapure water. The pain behavioral test proceeded for at least 10 min after CNO injection.

### 4.8. Statistical Analysis

Data are presented as original forms or means ± SEMs and were analyzed using GraphPad 8.0. Data were tested by unpaired Student *t*-test for two-group comparisons, or two-way ANOVA followed by Sidak’s multiple-comparison test for multiple comparisons. All data were stated individually in the Section 2. Statistical significance was set to *p* < 0.05.

## Data Availability

The original contributions presented in the study are included in the article/Supplementary Materials, further inquiries can be directed to the corresponding author/s.

## Supplementary Materials

The supporting information can be downloaded at: https://www.mdpi.com/article/10.3390/ijms25189774/s1.
