# Supplementary material for: The Role of the Mu Opioid Receptors of the Medial Prefrontal Cortex in the Modulation of Analgesia Induced by Acute Restraint Stress in Male Mice
Source: Int J Mol Sci. 2024 Sep 10;25(18):9774. doi: 10.3390/ijms25189774 (PMC11431787; doi:10.3390/ijms25189774)
Supplement: Supplementary file 1 [file ijms-25-09774-s001.zip › ijms-3151637-supplementary.pdf]

Table S1. Details of MOR-counting statistical data in MOR WT and MOR KO mice (related to figure 2)

MOR WT：

Mouse No.	neural cells	MOR positive cells	MOR positive cells /neural cells ratio
1	771	391	50.71%
2	803	398	49.57%
3	792	382	48.23%
Averaged			49.50%

MOR KO：

Mouse No.	neural cells	MOR positive cells	MOR positive cells /neural cells ratio
1	863	9	1.04%
2	811	5	0.62%
3	778	8	1.03%
Averaged			0.90%

Table S2. Details of MOR-counting statistical data in MORGlut WT and MORGlut KO mice (related to figure 3)

MORGlut WT：

Mouse No.	Glut-positive cells	MOR- and Glut-double positive cells	Double positive cells/Glut-positive cells
1	587	228	38.84%
2	595	203	34.12%
3	619	227	36.67%
Averaged			36.54%

MORGlut cKO：

Mouse No.	Glut-positive cells	MOR- and Glut-double positive cells	Double positive cells/Glut-positive cells
1	573	11	1.92%
2	544	19	3.49%
3	586	13	2.22%
Averaged			2.54%

Table S3. Details of MOR-counting statistical data in MORGABA WT and MORGABA KO mice (related to figure 4)

MORGABA WT：

Mouse No.	GABA-positive cells	MOR- and GABA-double positive cells	Double positive cells /GABA-positive cells
1	84	56	66.67%
2	77	49	63.64%
3	86	64	74.42%
Averaged			68.24%

MORGABA cKO：

Mouse No.	GABA-positive cells	MOR- and GABA-double positive cells	Double positive cells /GABA-positive cells
1	79	5	6.33%
2	65	4	6.15%
3	72	5	6.94%
Averaged			6.47%

Table S4. Details of fos-counting statistical data in mice under unstress or restraint stress exposure (related to figure 5)

Unstress：

Mouse No.	EYFP cells	c-fos positive EYFP cells	c-fos positive EYFP cells/EYFP cells
1	73	7	9.59%
2	51	3	5.88%
3	62	5	8.06%
Averaged			7.84%

Stress：

Mouse No.	EYFP cells	c-fos positive EYFP cells	c-fos positive EYFP cells/EYFP cells
1	55	26	47.27%
2	67	35	52.23%
3	50	26	52.00%

Averaged			50.50%

Table S5. Details of fos-counting statistical data in MORGABA WT and MORGABA KO mice mice under unstress or restraint stress exposure (related to figure 6)

MORGABA cKO Unstress：

Mouse No.	EYFP cells	c-fos positive EYFP cells	c-fos positive EYFP cells/EYFP cells
1	58	0	0
2	69	3	4.35%
3	61	0	0
Averaged			1.45%

MORGABA cKO Stress：

Mouse No.	EYFP cells	c-fos positive EYFP cells	c-fos positive EYFP cells/EYFP cells
1	59	0	0
2	66	3	4.55%
3	60	3	5.00%
Averaged			3.18%

MORGABA WT Unstress：

Mouse No.	EYFP cells	c-fos positive EYFP cells	c-fos positive EYFP cells/EYFP cells
1	68	8	11.76%
2	67	5	7.46%
3	54	3	5.56%
Averaged			6.41%

MORGABA WT Stress：

Mouse No.	EYFP cells	c-fos positive EYFP cells	c-fos positive EYFP cells/EYFP cells
1	66	27	40.91%
2	65	31	47.70%
3	63	33	52.38%
Averaged			46.99%
